# Optimization of a magnetic bead-based assay (MAGPIX^®^-Luminex) for immune surveillance of exposure to malaria using multiple *Plasmodium* antigens and sera from different endemic settings

**DOI:** 10.1186/s12936-018-2465-4

**Published:** 2018-09-06

**Authors:** Marie Louise Varela, Babacar Mbengue, Aissata Basse, Cheikh Loucoubar, Inès Vigan-Womas, Alioune Dièye, Aissatou Toure, Ronald Perraut

**Affiliations:** 1 Unité d’Immunologie, Institut Pasteur de Dakar, 36 Avenue Pasteur, BP 220, Dakar, Sénégal; 2 Unité d’Immuno-Génétique, Institut Pasteur de Dakar, 36 Avenue Pasteur, BP 220, Dakar, Sénégal; 3 G4 Biostatistiques Bioinformatique et Modélisation, Institut Pasteur de Dakar, 36 Avenue Pasteur, BP 220, Dakar, Sénégal; 4 Unité d’Immunologie des Maladies Infectieuses, Institut Pasteur de Madagascar, Antananarivo, Madagascar; 5 Département de Parasitologie et Insectes Vecteurs, Institut Pasteur, 26-28 Rue du Dr Roux, 75015 Paris, France

**Keywords:** Malaria, Biomarkers, Magnetic beads assay, Multiplex, IgG response

## Abstract

**Background:**

Serological markers are potentially useful tools for monitoring the progress of malaria control programs, but a better understanding of antibody response dynamics is necessary. The use of a magnetic bead-based immunoassay (MBA) is advantageous compared to ELISA, due to its multiplexing capacity, but limited information is available on the standardization and validation of this assay.

**Methods:**

Several parameters for multiplex testing of antibodies to *Plasmodium* antigens were analysed using a set of 4 antigens and 98 sera from Senegalese rural asymptomatic and urban symptomatic individuals. The 4 antigens included *Plasmodium falciparum* CSP and PfAMA1 peptides, recombinant *P. falciparum* MSP4p20 and a *Plasmodium malariae* CSP (PmCSP) peptide. Comparisons with ELISA were done using MSP4p20 and whole schizont extract (SE) antigens.

**Results:**

The use of fewer beads (1000 beads per well instead of 2000) and 5 µg of antigen per 10^6^ bead were validated as lower amounts. The use of a carrier protein (BSA) was shown to be critical when using peptides and the effect of a 24 h delayed measures was evaluated (5–25% signal decrease). Analysis of Ab responses showed almost equally high levels and prevalence in all transmission settings. Clear distinctions between rural and urban malaria were noted using PmCSP and SE antigens.

**Conclusions:**

This study underlines the importance of further optimization of the MBA technique and highlights the interest of using multistage/multispecies antigens for surveillance of malaria in endemic settings.

## Background

Malaria remains one of the world’s deadliest diseases with over 214 million clinical cases (range: 149–303 million) and an estimated 438,000 deaths from *Plasmodium falciparum* infection in 2016, primarily in young children from sub-Saharan Africa [1]. Substantial scale-up of integrated intervention strategies including artemisinin-based combination therapy (ACT), universal coverage with long-lasting insecticide-impregnated bed nets (LLINs), systematic diagnosis using rapid tests (RDTs) and intermittent preventive treatment in vulnerable target groups have considerably reduced the burden of malaria in many countries. More than a million lives have been saved from 2000 to 2014, most of them among children under 5 years old [2].

The overall decrease in malaria transmission has changed its epidemiology although with substantial differences depending on geographical setting. In Senegal, 96% of the population lives in an area potentially at risk with > 1 case per 1000 population [1], and morbidity has decreased over 80% since 2005. Currently malaria is responsible for 3.6% of fatalities in the overall population and < 5% in children under 5 years. However, the incidence is still high (> 25%) in the south east and in five other hotspot regions [3].

Ongoing efforts to achieve sustainable field results require strategies for accurate evaluation of malaria exposure to monitor the effectiveness of anti-malaria control measures. For this purpose, measurement of antibody responses to relevant immune markers can be used to evaluate exposure and/or immunity in exposed as well as in naïve populations [4, 5]. The immune control of *P. falciparum* infection is complex, and requires the combined action of antibodies and cell mediated immune responses against both pre-erythrocytic and blood stages [6]. Antibody responses occur against a broad array of antigens [7] correlating with susceptibility to clinical and severe malaria [6].

Importantly, where malaria transmission has fallen, asymptomatic malaria infections with low and sub-microscopic parasite densities remain prevalent and much of the parasite reservoir becomes undetectable by standard active and passive case detection [5, 8]. Therefore, effective mass screening and treatment campaigns will most likely need more sensitive assays, such as molecular based assays and/or multiplex antibody biomarkers to (i) evaluate the level of immunity that is potentially protective and, (ii) track live invisible circulating parasites. For such campaigns, the evaluation of immune status is important for measuring the impact of combined intervention strategies (including potential loss of immunity), which can be accurately reflected by the magnitude of antibody responses to relevant biomarkers [9].

Several studies have focused on the identification of reliable predictors for exposure [10–12], susceptibility to infection and potential occurrence of complications during malaria episodes [4, 8, 10, 13, 14]. Thus, use of antibody responses as markers can also help to optimize therapeutic strategies and reduce disease burden  [15, 16]. Serological techniques, including ELISA, the standard in biomedical assays, generally provide quantitative measurements of IgG to one antigen at a time. High throughput measurement of antibody responses to large panels of antigens (Ag) by multiplex assays opens new opportunities for serological investigation and prior studies have demonstrated the benefits of multiplex immunoassays for monitoring malaria [9, 17–20]. The magnetic bead-based assay (MBA) using Luminex^®^ beads, the Magpix^®^ device, and protocols assessed in the present work, has been used in Cambodia [21], the Ivory coast [22, 23] and in two Senegalese endemic villages [24, 25].

This paper achieves two distinct goals. The first is technical optimization of the assay and the second is to further validate assay performance by measuring antibody responses against four *Plasmodium* antigens using a panel of sera from different Senegalese settings. Following previous reports [26, 27], several parameters were investigated here including the coupling procedure, minimal use of beads for reliable analysis, plate stability for delayed data acquisition, use of unconjugated peptide antigens and comparison between ELISA and MBA for one antigen i.e. MSP4.

## Methods

### Study design, characteristics of sera tested

A retrospective serological study was conducted using a panel of 98 archived samples previously collected from the villages of Dielmo (holoendemic rural area), Ndiop (mesoendemic rural area), and from the Hôpital Principal de Dakar (HPD, urban area) for acute severe malaria (SM). In the rural endemic settings of Dielmo and Ndiop, individuals did not have symptomatic *P. falciparum* infection and did not show microscopic positive parasite carriage at the time of the sampling.

There was no significant difference (P > 0.05, Mann–Whitney test) in the age distribution of the different groups from rural and urban zones. Ethical approvals were obtained from the investigators’ institutions and the National Ethical Committee from Ministry of Health CNERS (Comité National d’Ethique pour la Recherche en Santé). Villagers from Dielmo (holoendemic area) and Ndiop (mesoendemic area) are involved in an ongoing longitudinal survey to study the acquisition and maintenance of natural immunity as described previously [28–30]. Informed written consent for inclusion in the survey was annually obtained from all participants or their parents or guardians on a voluntary consent form written in both French and in Wolof, the local language. The follow-up procedure included regular sampling [29].

For hospitalized individuals, sera used were those prescribed for medical biology during follow-up of treatment that were available after the end of processing by the hospital Biology Laboratory. An informed consent or assent for the use of withdrawal for research purpose was obtained from patients or their parents explained in both French and in Wolof, following the procedure already described [31].

### Antigens

The multiplex magnetic Luminex^®^ bead-based immunoassay using Magpix technology was assessed by analysing the IgG response to four *Plasmodium* antigens: (i) baculovirus recombinant *P. falciparum* MSP4p20 (NF54 allele), purified by metallo-affinity chromatography as described previously [32, 33]; (ii) PfAMA1 peptide: YKDEIKKEIERESKRIKLNDNDDEGNKKIIAPRIFISDDKDSLKC; (iii) PfCSP peptide: (NANP)_9_-NVDPNVDPC; (iv) PmCSP peptide: (NAAG)_9_-NDAGC. The peptides used in this study were designed as previously described [9], with an N-terminal cysteine residue added to allow unidirectional coupling to BSA by the manufacturer (GenScript HK Inc., Hong Kong, China, or Genecust, France). For comparisons, 2 non-conjugated peptides without the N-terminal cysteine residue (PfCSP and PfAMA1) were used. The purity of each peptide was estimated > 85% by HPLC and mass spectrometry.

The schizont extracts were prepared from 07/03 parasites (Dielmo strain adapted to culture) cultivated in candle jars in O + erythrocytes with 10% human serum. Schizonts concentrated to ∼ 95% were lysed in an equal volume of sterile, distilled water and kept at − 80 °C until use [34, 35].

### Covalent coupling of Ag to beads

The covalent coupling of PfMSP4p20 recombinant protein, BSA and the 5 peptides to carboxylated magnetic Luminex^®^ microspheres by the carbodiimide reaction (Luminexcorp, Austin, USA) was done using the xMAP^®^ Antibody Coupling Kit (ref 40-50016, Luminexcorp, Austin, USA) according to the manufacturers’ instructions. Briefly, 2.5 × 10^6^ beads were used in a working volume of 500 µL. All washing steps including buffer changes, bead centrifugation, vortexing and water-bath sonication for bead dispersal were done the manufacturers’ instructions. After the carbodiimidehypochloride (EDC) activation step, 5 µg of Ag per million beads was added to the activation buffer and mixed by rotation in the dark for 2 h. After centrifugation and washing steps, the supernatant was removed and replaced by 1 mL wash buffer and kept in the dark at 2–8 °C. The final bead count showed a mean recovery of 95% coupled beads. The coupled microspheres were kept in the washing/storage buffer at 4 °C in the dark until use.

### Immunoassay multiplex Magpix

The custom magnetic bead-based MAGPIX^®^-Luminex Assay (MBA), adapted to parallel the steps used in the standard ELISA technique has been previously described [24, 26, 27]. The mix of microspheres was kept in an opaque vial. 2.5 µL aliquots containing 1000 beads per Ag were dispensed to individual wells of a white polystyrene opaque round bottom microtiter plate (Ref.103977741, Fisher Scientific, Illkirch, France). 100 μL of plasma diluted 1/200 in PBS, 0.01% Tween, 1% BSA (PBSB) was added to duplicate wells, mixed and incubated protected from light at room temperature on a microplate shaker (IKA^®^MTS, Wilmington, NC) at 350 rpm for 45 min. After washing twice, 100 μL of phycoerythrin-labelled goat anti-human IgG diluted 1:500 (gamma-chain specific, F(ab`)_2_ fragment-R-phycoerythrin (Sigma, P-8047 St. Louis, MO) in PBSB was added and incubated 45 min in the dark with shaking at 350 rpm. The beads were then re-suspended in 120 μL PBSB and analyzed on a Multiplex MAGPIX system (Millipore, USA) using the xPONENT 4.1 software for data acquisition. Antibody responses were expressed in median fluorescence intensity (MFI) per sample as stated by manufacturer’s instructions; readings were considered positive when the signal was greater than the mean MFI signal + 3 SD of 6 naïve control sera (a pool of non-immune sera from blood donors living in France).

### Elisa

The MSP4p20 and schizont extract antigens were coated on Nunc MaxiSorp^®^ plates (eBioscience). Sera to be tested were diluted at a 1/200. All other procedures were as described [24, 34–36]. Each plate included two positive controls: a pool of human immune IgG (kind gift from Prof M. Hommel) and a pool of 30 sera collected from clinically highly immune adults from Dielmo. The negative naïve control was a pool of non-immune sera from blood donors living in France. For inter-assay comparisons, results were expressed as absorbance ratios corresponding to OD-sample/OD-naïve. Positive responders were individuals with an absorbance ratio over 2 corresponding to the mean OD of naïve controls + 3 SD.

### Statistical analysis

Comparisons for categorical variables were done using the Fisher exact test, continuous variables of antibody responses were analysed using the Kruskal–Wallis, Wilcoxon and the Spearman rank correlation test for non-normally distributed data. Statistical analysis was performed using R and Statview 5.0 software.

## Results

### Optimization of multiplex protocols

The first aim of this study was the technical optimization of the protocol, including parameters such as the optimal number of beads per well, the importance of BSA-conjugated peptides and proteins, impact of delayed reading on fluorescence intensity.

#### Reduction in bead numbers: 1000 vs 2000 beads per well per Ag

The use of 5 µg of Ag per million beads was found largely sufficient here, as confirmed by further recognition of Ag coated beads by positive controls and sera. In addition, the coupling procedure as already described [24] led to minimal loss of beads (< 10% for all antigens). The standard number of beads per well for each Ag throughout the optimization process was 2000 as initially determined [24].

**Fig. 1:**
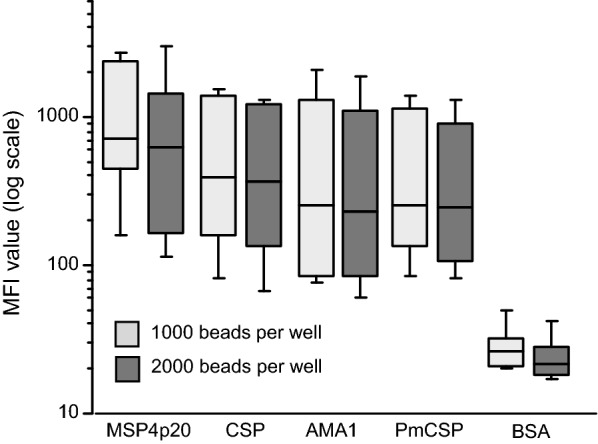
Comparison of results using reduced concentration of beads: 1000 *vs* 2000 beads. In this figure is shown as boxplot the levels of Ab responses measured when using 1000 or 2000 beads per well. No significant difference was evidenced between signals measured with the two procedures

To check whether similar results could be obtained with fewer beads, a comparison of MFI values obtained with 2000 beads/Ag/well and 1000 beads/Ag/well was done on a set of 30 sera from an endemic area with positive and negative controls. As shown on Fig. 1, MFI values for all antigens tested were similar without significant differences between measures (P = 0.18, by Friedman repeated measures analysis of variance on ranks) and included a low background with BSA as the negative control. The final recovery of beads for readout by the Magpix devices was more than sufficient in both cases i.e. a mean number of 250 and 480 beads per well for initial quantities of 1000 and 2000 beads, respectively. This is well above the minimum number of 50 beads required for relevant statistical measures. This optimisation allows for bead economy using the MBA and the current standard protocol is 1000 beads per well per Ag as the starting material.

#### Plate conservation

**Fig. 2:**
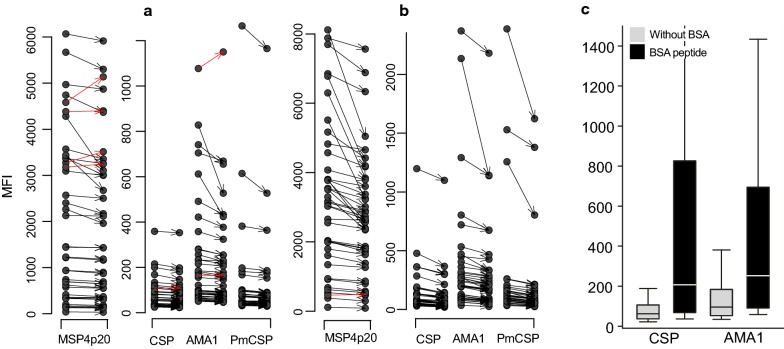
Effect of plate conservation 24 h at 4 °C (**a**), 24 h at room temperature (**b**) and impact of the absence of BSA carrier for peptides conjugation to beads (**c**). This figure shows the effect of delayed acquisition of MFI signal after 24 h at 4 °C (**a**) and 24 h at room temperature (**b**). Individual antibody responses against the different antigens are shown as dot blot and arrow to the corresponding delayed measure. A red arrow links particular individual measures that did not decrease. The decrease of level was significant (P < 10^−3^, Wilcoxon signed rank test for paired data) ranging from 5% to 16% after 24 h at 4 °C, and from 17% to 25% after 24 h at RT. **c** shows the comparison of antibody responses against CSP and PfAMA1 peptides with (black) or without BSA (light grey); levels of responses are significantly lower without BSA (P < 10^−3^, Wilcoxon signed rank test)

One important question involves plate stability and allowance for delay in data acquisition using the Magpix device. To evaluate the effect of delayed data acquisition, plates from two subsets of sera were incubated for 24 h either at 4 °C or room temperature (n = 40 sera each) following a standard reading and then read a second time given the fact that there was sufficient remaining amount of beads for second run. The comparison of data measured before and after the 24 h delay shows a similar significant reduction of MFI (P < 10^−3^, Wilcoxon signed rank test) in all cases. Decreases ranged from 5% to 16% after 24 h at 4 °C (Fig. 2a), and from 17% to 25% after 24 h at RT (Fig. 2b).

#### The importance of BSA carrier for conjugation of peptides to beads

The requirement for BSA as a carrier for conjugation of peptides to the beads was tested with two unconjugated peptides (CSP and PfAMA1) on a large subset of sera (n = 160) from immune individuals living in holoendemic or mesoendemic transmission areas. Figure 2c shows highly significant reduced responses (8 and 4 times lower for CSP and PfAMA1, respectively). Despite a significant correlation between responses against peptides with or without BSA (Spearman rank test, P < 10^−3^, r = 0.61 and 0.82 for CSP and PfAMA1, respectively), the results obtained without BSA conjugation were uninterpretable due to the low levels of MFI.

### Comparison of IgG responses measured by MBA and ELISA

**Fig. 3:**
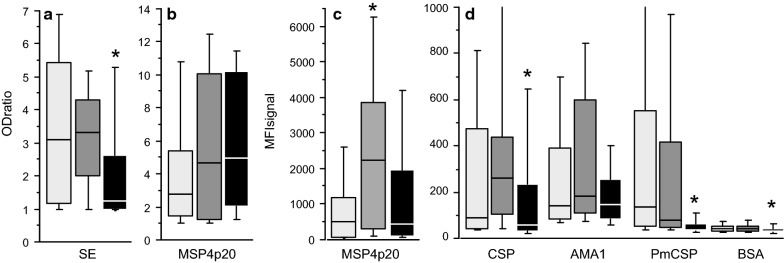
Comparison of the distribution of antibody responses in three different settings. Antibody responses in individuals from Dielmo (light grey), Ndiop (dark grey) and urban hospitalized (black) are plotted as boxplots. OD ratio to SE, MSP4p20 were shown in panel (**a** and **b)**, MFI responses to PfMSP4p20 and other antigens included in this study were plotted in (**c** and **d)**. Asterisks indicate significant lower levels of IgG to SE, PfCSP, PmCSP and BSA in urban hospitalized, and higher level of Ab to PfMSP4p20 in Ndiop when measured by MBA

Antibody responses were measured on a set of 98 sera originating from the three different endemic areas: 25 samples from Dielmo (mean age 23.2, range 3–72.5 yo), 37 samples from Ndiop (mean age 26.5, range 5.1–58.3 yo) and 36 samples from SM patients (mean age 28.2, range 12–63 yo) were tested. MBA analysis was done using the MSP4p20, PfAMA1 and CSP Ag from *P. falciparum* and CSP from *P. malariae* (PmCSP). The ELISA standard assay was done using (i) whole shizont extract (SE) as a reference for anti-*Plasmodium* IgG response and; (ii) MSP4p20 antigen to compare ELISA and MBA readouts. Results of antibody responses and prevalence of responders are given in Table 1 and illustrated in Fig. 3. Prevalence of antibody responses ranged from 19 to 84% with different profiles according to the Ag tested.

**Table 1:** Seroprevalence and IgG level of response

Antigen	Dielmo		Ndiop		Urban SM	
	IgG level	Prev^c^ (%)	IgG levels	Prev (%)	IgG levels	Prev (%)
MSP4-p20^a^	1103	64	2684	84	1315	69
[Range]	[28–10,580]		[42–13,160]		[23–6381]	
PfCSP^a^	262	52	536	57	187	47
[Range]	[22–985]		[22–4969]		[18–1061]	
PfAMA1^a^	282	52	402	49	456	58
[Range]	[12–2352]		[43–3065]		[46–8930]	
PmCSP^a^	691	56	537	62	79	19
[Range]	[28–7365]		[29–9278]		[23–991]	
MSP4-p20^b^	4.5	56	5.9	70	6.0	75
[Range]	[1–14.7]		[1–15.3]		[1–12.9]	
Schizont Ag^b^	3.5	64	3.1	76	2.2	36
[Range]	[1–9.3]		[1–6.0]		[1–7.4]	

^a^ Levels and prevalence of Ab responses measured by MBA (expressed in MFI)

^b^ Levels and prevalence of Ab responses measured by ELISA (expressed in OD ratio)

^c^ *Prev* prevalence of responders

The MBA results show the highest Ab levels and prevalence for MSP4p20 in all groups compared to the other antigens tested. Almost no differences in levels and prevalence of PfAMA1 and PfCSP were observed, except for SM with significantly lower levels compared to Ndiop (P = 0.03). As shown in Fig. 3, antibody responses to the control carrier BSA were extremely low especially for urban malaria, further validating the use of BSA as a carrier for conjugation of peptide Ag biomarkers.

**Fig. 4:**
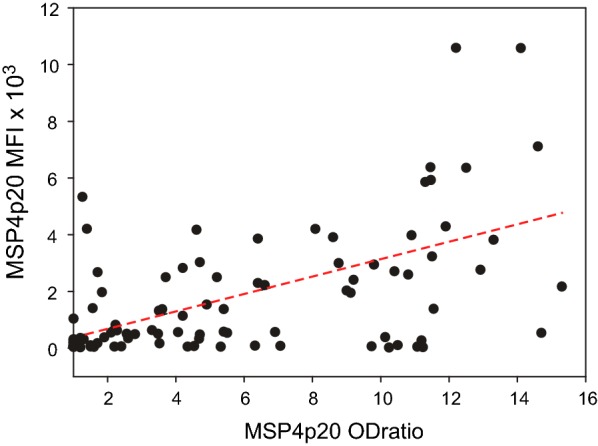
Correlation between OD ratio and MFI measures for MSP4p20. ELISA OD ratio (X axis) *vs* MFI values (Y axis) values from all 98 samples are plotted for IgG responses against MSP4p20 (b). The linear regression line is indicated, a significant correlation (P < 10^−3^) of antibody responses measured by two methods was evidenced (rho = 0.52 by Spearman rank test; r = 0.48 by linear regression)

Comparing ELISA *vs* MBA analyses showed no significant difference between groups for MSP4p20 by ELISA, but a slightly significant lower level of response in Ndiop when measured by MBA (Table 2). Correlation between OD ratio and MFI values to MSP4p20 for all samples was significantly positive (r = 0.52, P < 10^−4^; Spearman test) (Fig. 4). However, when each group were considered separately, correlation was strong and significant for Dielmo and Ndiop (r = 0.76 and 0.85, P < 10^−2^, respectively) but not for SM.

**Table 2:** Systematic comparison of levels and prevalence of antibody responses between the different settings

	Dielmo *vs* Ndiop		Dielmo *vs* SM		Ndiop *vs* SM	
	Level^a^	Prev^b^	Level	Prev	Level	Prev
Schiz. Extr.	NS^c^	NS	0.03	0.04	0.004	< 10^−2^
MSP4 OD ratio	NS	NS	NS	NS	NS	NS
MSP4mfi	0.048	NS	NS	NS	0.01	NS
PfCSPmfi	NS	NS	NS	NS	0.03	NS
PfAMA1mfi	NS	NS	NS	NS	NS	NS
PmCSPmfi	NS	NS	< 10^−3^	0.005	< 10^−3^	< 10^−2^
BSAmfi	NS	NA	0.01	NA	0.004	NA

^a^ Statistical comparison of levels of Ab responses (Mann–Whitney test)

^b^ Comparison of prevalence of responders (Fisher exact test)

^c^*NS* non significant, *NA* non available, *SM* severe malaria

Regarding differences between urban and rural endemic sites, Ab levels and prevalence to SE and PmCSP were significantly lower in SM compared to Dielmo and Ndiop (Fig. 3), indicating the increased exposure of the villagers to both *P. falciparum* and *P. malariae* transmission.

### Correlations between Ab responses, age of individuals and different antigens

There was a significant (P < 10^−2^) age-related correlation of antibody responses for the 98 samples: r = 0.32 to 0.58 (PfAMA1 < PmCSP < SE < MSP4p20 < PfCSP) which was stronger for Dielmo and Ndiop, ranging from 0.62 to 0.8 (except PfAMA1 in Ndiop), but was not significant for SM (except PfAMA1: r = 0.41, P = 0.01). Correlations between Ab responses to different antigens was analysed by reciprocal spearman rank test. For Dielmo and Ndiop, correlations were strong (r = 0.41 to 0.85) and significant (P < 10^−3^) except for PfAMA1. In SM samples, significant correlation was only between PfAMA1, PfCSP and MSP4p20 (r = 0.57 to 0.67, P < 10^−3^).

## Discussion

In the context of malaria pre-elimination, the profiling of malaria immunity has become a tool for monitoring the ongoing efficiency of various interventions. Evaluation of malaria exposure and monitoring of anti-malaria control measures are necessary for long-term effective field results [4, 5]. The commonly available techniques in malaria sero-epidemiology such as enzyme-linked immunosorbent assay (ELISA) have proven specificity and sensitivity against pre-erythrocytic and blood stages of the parasite. However, large scale evaluation of multiple antigen targets using monoplex assays, such as ELISA require substantial volumes of sample, is labour intensive and remains relatively expensive and time consuming when testing over 6–8 antigens.

Multiplex assays are based on the same principles as ELISA but overcomes its limitations, requiring both less sample and antigen. A convenient recent multiplex approach is the compact Luminex/MAGPIX^®^ system using colour-coded magnetic beads, displayed by magnet in a monolayer and based on LED/CCD imaging technology. This platform considerably reduces the costs of the multiplex approach and has proven to be robust when used in different countries/laboratories for malaria, [21, 22] as well as recently for viral surveillance [37, 38].

Multiplex assays have an increasing number of applications, but they usually require preliminary protocol validation by comparison with ELISA, as illustrated for measuring cytokine levels [39] in several commercial ready-to-use assays. However, analysis of antibody responses to the numerous *Plasmodium* parasite antigens is a complex procedure often lacking standardization. For example, methods for measuring Ab responses and the expression of indirect ELISA results vary in different laboratories, including substantial differences in methods for end-point titration calculations [40]. Similarly, MBA assays require protocol optimization and standardization as indicated by the increasing number of reports related to the use of Luminex beads [9, 14, 19] and the Magpix system [24–27].

Several important points emerge from the analyses presented here. First the standard amount of antigen used for coupling seems in excess and could likely be reduced, but should be confirmed for each antigen tested. For peptide biomarkers, the carrier (BSA here) was confirmed to be essential for optimal recognition after coupling to beads, including for long peptides over 40 amino acids long (PfCSP and PfAMA1). Unconjugated peptides are probably tightly coupled to the beads, thus inhibiting epitope recognition and causing signal reduction. In addition, the use of 1000 beads per antigen per well was largely sufficient giving similar results with 2000 or more beads [14, 26]. Second, it was showed that plates can be read 24 h after being kept at 4 °C or at RT but with substantial reduction of signal depending on antigen tested. This point is important because each plate requires almost 1 h for 96 well complete reading. Data normalization using a positive control is under investigation to address this point. Finally, the current protocol of coupling 5 µg of antigen per million beads and using 1000 beads per test reduces substantially the cost of the MBA without affecting the quality of the results.

MBA antibody responses were measured in sera from three different transmission areas using peptides derived from *P. falciparum* (PfCSP, PfAMA1) and *P. malariae* (PmCSP) and a recombinant *P. falciparum* protein (MSP4p20). There were several observations of interest.

First, BSA controls showed negligible background signals for SM patients, and as previously observed for the endemic villagers [24]. Prevalence and levels of antibodies to both *P. falciparum* peptides was high, with only minor differences between clinically immune villagers at endemic sites and urban symptomatic hospitalized patients. Only Ab responses to PfCSP were slightly lower in SM compared to Ndiop confirming these Ag as relevant markers for monitoring malaria transmission intensity [41].

Second, *P. malariae* CSP was the only peptide antigen that significantly differentiated urban patients and endemic villagers including prevalence and intensity of responses (Table 2). This supports the relevance of this marker for monitoring *P. malariae* circulation as indicated previously in Dielmo and Ndiop [42]. Thus, there appears to be long half-life sero-conversion for PmCSP [26], which showed substantial unexpected positivity in high endemic areas [22].

Third, the MSP4p20 antigen was used because it is a strongly recognized marker of *P. falciparum* infection [22, 23, 43], with a prevalence of over 80% in Dielmo and Ndiop (unpublished results). Therefore, this antigen was simultaneously tested by ELISA and MBA and showed a significant correlation between these two methods. Comparable correlates were reported in Ndiop for MSP1 [24], and for several other antigens in this seasonal transmission area [14]. Here there were lower correlation coefficients calculated with all sera i.e. SM and asymptomatic villagers compared to results calculated separately with samples from each site. For Ndiop, correlations were comparable to results reported for MSP1, despite the limited number of samples tested [44]. Discrepancies were especially apparent when analysing antibody responses in CM samples. The profile of antibody responses for low endemic urban malaria was substantially different, with no relationship between age and response level as already observed in this setting [45]. Such profiles reflect the limited cumulative individual exposition in urban areas, in agreement with the observed antigen dependent longevity of antibody responses which is independent of response magnitude [46]. Antibody responses to MSP4p20 were equally strong in all settings as opposed to those for schizont extract and *P. malariae*. Interestingly, SE appears to be a relevant differential indicator of cumulative immunity, in addition of antibody response to *P. malariae* that rather reflect cumulative rural transmission profile. However, SE is an undefined mixture of antigens requiring further standardization [34, 35] and is not presently usable for the MBA technique. In longitudinal and cross-sectional studies, the use of crude extract as a basic marker of parasite exposure remains a valid observation. SE was used to demonstrate the decay of antibody responses after implementation of control measures in community investigations [36], and is used as a reference for comparison with responses to defined target antigens [14, 44].

Taken together, these technical details contribute to the usefulness of MBA, especially for multiple, simultaneous analyses of antibody responses with small sample volumes. In particular, the potential of MBA for use with dried blood spot samples stored at 4 °C is an attractive alternative for large-scale serological studies [4]. Multiplex assays using blood spots permitted very large-scale analysis of seroconversion rates and *Plasmodium* transmission intensity in low-endemic populations [21, 26]. However, results were unclear: on one side MBA provided a higher titration capacity than ELISA using eluted IgG from dried spots [47]; on the other side antibody levels for both PfAMA1 and MSP1 were found lower than ELISA (11.3–21.4%) limiting its capacity to distinguish age-related sero-prevalence patterns [48]. Finally, the use of blood spots merits further investigation to elaborate accurate scaling protocols.

## References

[1] WHO (2017). World Malaria Report 2017.

[2] WHO (2014). World Malaria Report 2017.

[3] PNLP. Activity report year 2014. Ministry of Health and Social Education (National Programme against Malaria) 2015, July 2015.

[4] Corran P, Coleman P, Riley E, Drakeley C (2007). Serology: a robust indicator of malaria transmission intensity?. Trends Parasitol.

[5] Cotter C, Sturrock HJ, Hsiang MS, Liu J, Phillips AA, Hwang J (2013). The changing epidemiology of malaria elimination: new strategies for new challenges. Lancet.

[6] Riley EM, Stewart VA (2013). Immune mechanisms in malaria: new insights in vaccine development. Nat Med.

[7] Osier FH, Fegan G, Polley SD, Murungi L, Verra F, Tetteh KK (2008). Breadth and magnitude of antibody responses to multiple *Plasmodium falciparum* merozoite antigens are associated with protection from clinical malaria. Infect Immun.

[8] Sarr JB, Orlandi-Pradines E, Fortin S, Sow C, Cornelie S, Rogerie F (2011). Assessment of exposure to *Plasmodium falciparum* transmission in a low endemicity area by using multiplex fluorescent microsphere-based serological assays. Parasit Vectors.

[9] Ambrosino E, Dumoulin C, Orlandi-Pradines E, Remoue F, Toure-Balde A, Tall A (2010). A multiplex assay for the simultaneous detection of antibodies against 15 *Plasmodium falciparum* and *Anopheles gambiae* saliva antigens. Malar J.

[10] Akpogheneta OJ, Duah NO, Tetteh KK, Dunyo S, Lanar DE, Pinder M (2008). Duration of naturally acquired antibody responses to blood-stage *Plasmodium falciparum* is age dependent and antigen specific. Infect Immun.

[11] Badu K, Gyan B, Appawu M, Mensah D, Dodoo D, Yan G (2015). Serological evidence of vector and parasite exposure in Southern Ghana: the dynamics of malaria transmission intensity. Parasit Vectors.

[12] Badu K, Siangla J, Larbi J, Lawson BW, Afrane Y, Ong’echa J (2012). Variation in exposure to Anopheles gambiae salivary gland peptide (gSG6-P1) across different malaria transmission settings in the western Kenya highlands. Malar J.

[13] Cook J, Kleinschmidt I, Schwabe C, Nseng G, Bousema T, Corran PH (2011). Serological markers suggest heterogeneity of effectiveness of malaria control interventions on Bioko Island, equatorial Guinea. PLoS ONE.

[14] Ondigo BN, Hodges JS, Ireland KF, Magak NG, Lanar DE, Dutta S (2014). Estimation of recent and long-term malaria transmission in a population by antibody testing to multiple *Plasmodium falciparum* antigens. J Infect Dis.

[15] Osier FH, Mackinnon MJ, Crosnier C, Fegan G, Kamuyu G, Wanaguru M (2014). New antigens for a multicomponent blood-stage malaria vaccine. Sci Transl Med.

[16] van den Hoogen LL, Griffin JT, Cook J, Sepulveda N, Corran P, Conway DJ (2015). Serology describes a profile of declining malaria transmission in Farafenni, The Gambia. Malar J.

[17] Cham GK, Kurtis J, Lusingu J, Theander TG, Jensen AT, Turner L (2008). A semi-automated multiplex high-throughput assay for measuring IgG antibodies against *Plasmodium falciparum* erythrocyte membrane protein 1 (PfEMP1) domains in small volumes of plasma. Malar J.

[18] Dodoo D, Atuguba F, Bosomprah S, Ansah NA, Ansah P, Lamptey H (2011). Antibody levels to multiple malaria vaccine candidate antigens in relation to clinical malaria episodes in children in the Kasena-Nankana district of Northern Ghana. Malar J.

[19] Fouda GG, Leke RF, Long C, Druilhe P, Zhou A, Taylor DW (2006). Multiplex assay for simultaneous measurement of antibodies to multiple *Plasmodium falciparum* antigens. Clin Vaccine Immunol.

[20] Proietti C, Verra F, Bretscher MT, Stone W, Kanoi BN, Balikagala B (2013). Influence of infection on malaria-specific antibody dynamics in a cohort exposed to intense malaria transmission in northern Uganda. Parasite Immunol.

[21] Kerkhof K, Sluydts V, Willen L, Kim S, Canier L, Heng S (2016). Serological markers to measure recent changes in malaria at population level in Cambodia. Malar J.

[22] Koffi D, Toure AO, Varela ML, Vigan-Womas I, Beourou S, Brou S (2015). Analysis of antibody profiles in symptomatic malaria in three sentinel sites of Ivory Coast by using multiplex, fluorescent, magnetic, bead-based serological assay (MAGPIX). Malar J.

[23] Koffi D, Varela ML, Loucoubar C, Beourou S, Vigan-Womas I, Toure A (2017). Longitudinal analysis of antibody responses in symptomatic malaria cases do not mirror parasite transmission in peri-urban area of Côte d’Ivoire between 2010 and 2013. PLoS ONE.

[24] Perraut R, Richard V, Varela ML, Trape JF, Guillotte M, Tall A (2014). Comparative analysis of IgG responses to *Plasmodium falciparum* MSP1p19 and PF13-DBL1alpha1 using ELISA and a magnetic bead-based duplex assay (MAGPIX(R)-Luminex) in a Senegalese meso-endemic community. Malar J.

[25] Perraut R, Varela ML, Loucoubar C, Niass O, Sidibe A, Tall A (2017). Serological signatures of declining exposure following intensification of integrated malaria control in two rural Senegalese communities. PLoS ONE.

[26] Kerkhof K, Canier L, Kim S, Heng S, Sochantha T, Sovannaroth S (2015). Implementation and application of a multiplex assay to detect malaria-specific antibodies: a promising tool for assessing malaria transmission in Southeast Asian pre-elimination areas. Malar J.

[27] Perraut R, Varela ML, Mbengue B, Guillotte M, Mercereau-Puijalon O, Vigan-Womas I (2015). Standardization of a multiplex magnetic bead-based assay for simultaneous detection of IgG to Plasmodium antigens. J Immunol Tech Infect Dis.

[28] Trape JF, Rogier C (1996). Combating malaria morbidity and mortality by reducing transmission. Parasitol Today.

[29] Trape JF, Rogier C, Konate L, Diagne N, Bouganali H, Canque B (1994). The Dielmo project: a longitudinal study of natural malaria infection and the mechanisms of protective immunity in a community living in a holoendemic area of Senegal. Am J Trop Med Hyg.

[30] Trape JF, Tall A, Sokhna C, Ly AB, Diagne N, Ndiath O (2014). The rise and fall of malaria in a West African rural community, Dielmo, Senegal, from 1990 to 2012: a 22 year longitudinal study. Lancet Infect Dis.

[31] Mbengue B, Niang B, Niang MS, Varela ML, Fall B, Fall MM (2016). Inflammatory cytokine and humoral responses to *Plasmodium falciparum* glycosylphosphatidylinositols correlates with malaria immunity and pathogenesis. Immun Inflamm Dis.

[32] Bonnet S, Petres S, Holm I, Fontaine T, Rosario S, Roth C (2006). Soluble and glyco-lipid modified baculovirus *Plasmodium falciparum* C-terminal merozoite surface protein 1, two forms of a leading malaria vaccine candidate. Vaccine.

[33] Holm I, Nato F, Mendis KN, Longacre S (1997). Characterization of C-terminal merozoite surface protein-1 baculovirus recombinant proteins from *Plasmodium vivax* and *Plasmodium cynomolgi* as recognized by the natural anti-parasite immune response. Mol Biochem Parasitol.

[34] Diop F, Diop G, Niang M, Diouf B, Ndiaye D, Richard V (2015). The value of local malaria strains for serological studies: local strains versus Palo Alto reference strain. Malar J.

[35] Perraut R, Guillotte M, Drame I, Diouf B, Molez JF, Tall A (2002). Evaluation of anti-*Plasmodium falciparum* antibodies in Senegalese adults using different types of crude extracts from various strains of parasite. Microbes Infect.

[36] Diop F, Richard V, Diouf B, Sokhna C, Diagne N, Trape JF (2014). Dramatic declines in seropositivity as determined with crude extracts of *Plasmodium falciparum* schizonts between 2000 and 2010 in Dielmo and Ndiop, Senegal. Malar J.

[37] O’Hearn AE, Voorhees MA, Fetterer DP, Wauquier N, Coomber MR, Bangura J (2016). Serosurveillance of viral pathogens circulating in West Africa. Virol J.

[38] Satterly NG, Voorhees MA, Ames AD, Schoepp RJ (2017). Comparison of MagPix assays and enzyme-linked immunosorbent assay for detection of hemorrhagic fever viruses. J Clin Microbiol.

[39] Elshal MF, McCoy JP (2006). Multiplex bead array assays: performance evaluation and comparison of sensitivity to ELISA. Methods.

[40] Miura K, Orcutt AC, Muratova OV, Miller LH, Saul A, Long CA (2008). Development and characterization of a standardized ELISA including a reference serum on each plate to detect antibodies induced by experimental malaria vaccines. Vaccine.

[41] Wong J, Hamel MJ, Drakeley CJ, Kariuki S, Shi YP, Lal AA (2014). Serological markers for monitoring historical changes in malaria transmission intensity in a highly endemic region of Western Kenya, 1994–2009. Malar J.

[42] Roucher C, Rogier C, Sokhna C, Tall A, Trape JF (2014). A 20-year longitudinal study of *Plasmodium ovale* and *Plasmodium malariae* prevalence and morbidity in a West African population. PLoS ONE.

[43] Wang L, Richie TL, Stowers A, Nhan DH, Coppel RL (2001). Naturally acquired antibody responses to *Plasmodium falciparum* merozoite surface protein 4 in a population living in an area of endemicity in Vietnam. Infect Immun.

[44] Perraut R, Marrama L, Diouf B, Sokhna C, Tall A, Nabeth P, Trape JF (2005). Antibodies to the conserved C-terminal domain of the *Plasmodium falciparum* merozoite surface protein 1 and to the merozoite extract and their relationship with in vitro inhibitory antibodies and protection against clinical malaria in a Senegalese village. J Infect Dis.

[45] Mbengue B, Sylla Niang M, Ndiaye Diallo R, Diop G, Thiam A, Ka O (2015). IgG responses to candidate malaria vaccine antigens in the urban area of Dakar (Senegal): evolution according to age and parasitemia in patients with mild symptoms. Bull Soc Path Exot.

[46] Elliott SR, Fowkes FJ, Richards JS, Reiling L, Drew DR, Beeson JG (2014). Research priorities for the development and implementation of serological tools for malaria surveillance. F1000Prime Rep.

[47] Rogier E, Wiegand R, Moss D, Priest J, Angov E, Dutta S (2015). Multiple comparisons analysis of serological data from an area of low *Plasmodium falciparum* transmission. Malar J.

[48] Baidjoe A, Stone W, Ploemen I, Shagari S, Grignard L, Osoti V (2013). Combined DNA extraction and antibody elution from filter papers for the assessment of malaria transmission intensity in epidemiological studies. Malar J.

